# Diabetes Mellitus and the Risk of Alzheimer’s Disease: A Nationwide Population-Based Study

**DOI:** 10.1371/journal.pone.0087095

**Published:** 2014-01-29

**Authors:** Chin-Chou Huang, Chia-Min Chung, Hsin-Bang Leu, Liang-Yu Lin, Chun-Chih Chiu, Chien-Yi Hsu, Chia-Hung Chiang, Po-Hsun Huang, Tzeng-Ji Chen, Shing-Jong Lin, Jaw-Wen Chen, Wan-Leong Chan

**Affiliations:** 1 Department of Medical Research and Education, Taipei Veterans General Hospital, Taipei, Taiwan, R.O.C; 2 Division of Cardiology, Department of Medicine, Taipei Veterans General Hospital, Taipei, Taiwan, R.O.C; 3 Healthcare and Management Center, Taipei Veterans General Hospital, Taipei, Taiwan, R.O.C; 4 Division of Endocrinology and Metabolism, Department of Medicine, Taipei Veterans General Hospital, Taipei, Taiwan, R.O.C; 5 Department of Family Medicine, Taipei Veterans General Hospital, Taipei, Taiwan, R.O.C; 6 Cardiovascular Research Center, National Yang-Ming University, Taipei, Taiwan, R.O.C; 7 Faculty of Medicine, National Yang-Ming University, Taipei, Taiwan, R.O.C; 8 Institute of Pharmacology, National Yang-Ming University, Taipei, Taiwan, R.O.C; 9 Institute of Clinical Medicine, National Yang-Ming University, Taipei, Taiwan, R.O.C; 10 Institute of Hospital and Health Care Administration, National Yang-Ming University, Taipei, Taiwan, R.O.C; 11 Division of Cardiology, Department of Medicine, Zhudong Veterans Hospital, HsinChu, Taiwan, R.O.C; 12 Environment-Omics-Disease Research Centre, China Medical University Hospital, Taichung, Taiwan, R.O.C; 13 Graduate Institute of Clinical Medical Science, China Medical University, Taichung, Taiwan, R.O.C; University of Michigan Medical School, United States of America

## Abstract

**Objectives:**

Possible association between diabetes mellitus (DM) and Alzheimer’s disease (AD) has been controversial. This study used a nationwide population-based dataset to investigate the relationship between DM and subsequent AD incidence.

**Methods:**

Data were collected from Taiwan’s National Health Insurance Research Database, which released a cohort dataset of 1,000,000 randomly sampled people and confirmed it to be representative of the Taiwanese population. We identified 71,433 patients newly diagnosed with diabetes (age 58.74±14.02 years) since January 1997. Using propensity score, we matched them with 71,311 non-diabetic subjects by time of enrollment, age, gender, hypertension, hyperlipidemia, and previous stroke history. All the patients were followed up to December 31, 2007. The endpoint of the study was occurrence of AD.

**Results:**

Over a maximum 11 years of follow-up, diabetic patients experienced a higher incidence of AD than non-diabetic subjects (0.48% vs. 0.37%, *p*<0.001). After Cox proportional hazard regression model analysis, DM (hazard ratio [HR], 1.76; 95% confidence interval [CI], 1.50–2.07, *p*<0.001), age (HR, 1.11; 95% CI, 1.10–1.12, *p*<0.001), female gender (HR, 1.24; 95% CI, 1.06–1.46, *p = *0.008), hypertension (HR, 1.30; 95% CI, 1.07–1.59, *p = *0.01), previous stroke history (HR, 1.79; 95% CI, 1.28–2.50, *p*<0.001), and urbanization status (metropolis, HR, 1.32; 95% CI, 1.07–1.63, *p* = 0.009) were independently associated with the increased risk of AD. Neither monotherapy nor combination therapy with oral antidiabetic medications were associated with the risk of AD after adjusting for underlying risk factors and the duration of DM since diagnosis. However, combination therapy with insulin was found to be associated with greater risk of AD (HR, 2.17; 95% CI, 1.04–4.52, *p* = 0.039).

**Conclusion:**

Newly diagnosed DM was associated with increased risk of AD. Use of hypoglycemic agents did not ameliorate the risk.

## Introduction

Alzheimer’s disease (AD) is the most common neurodegenerative disease worldwide. With the increasing prevalence of AD, more and more people are becoming interested in identifying their risk of developing it [1]. AD causes a huge economic burden in worldwide [2]. There is no cure for the disease, which becomes progressively worse and leads eventually to death [3]. Although other major causes of death have been on the decrease, deaths from AD have been rising dramatically [4], [5]. The median survival from initial diagnosis is only 3.1 years for subjects with probable AD and 3.5 years for subjects with possible AD [5]. In Taiwan, AD is the most common cause of dementia [6]. Therefore, it is important to identify possible risk factors for AD, hoping these will point to effective prevention strategies for patients at risk.

Although some epidemiologic studies have shown that vascular risk factors are related to increased risk of AD, the cause and progression of AD are not well understood [7]. AD is characterized by pathological hallmarks in the brain, i.e., abnormal protein deposits (β-amyloid peptides) and τ-protein fibers (neurofibrillary tangles). To date, numerous studies have attempted to delineate risk factors for development and progression of AD, generating abundant theories on potential risk factors, preventive measures, and therapies. Recent studies have raised the possibility of a connection between diabetes mellitus (DM) and AD. Although some studies have found a higher risk of developing AD in diabetic patients [8]–[10], the association has been inconsistent [11]–[13]. Furthermore, the possible impact of hypoglycemic agents on the development of AD has also been unclear. We have therefore conducted a nationwide population-based study using the Taiwan National Health Insurance Research Database (NHIRD) to investigate the relationship between DM and subsequent AD incidence. We have also examined the possible impacts of hypoglycemic agents on the prevention of AD in diabetic patients.

## Materials and Methods

### Database

The National Health Insurance program in Taiwan has operated since 1995 and enrolls nearly all the inhabitants of Taiwan (21,869,478 beneficiaries out of 22,520,776 inhabitants at the end of 2002) [14]. Currently, the NHIRD at the National Health Research Institutes in Miaoli (Taiwan) has charge of the complete National Health Insurance claims database and has published several dozen extracted datasets for researchers. The National Health Research Institutes has released a cohort dataset made of 1,000,000 people who were alive in 2000 and has collected all records on these individuals from 1995 onward. These random samples have been confirmed by the National Health Research Institutes to be representative of the Taiwanese population. In this cohort dataset, each patient’s original identification number has been encrypted to protect privacy. But the encrypting procedure is consistent, so that the linkage of claims belonging to the same patient is feasible within the NHIRD. This study was exempt from full review by the Institutional Review Board, since the dataset used consisted of de-identified secondary data released to the public for research purposes.

### Study Patients

This study was conducted with the NHIRD, in which the diagnosis was supposed to be confirmed clinically by the individual physicians in charge for insurance claim purposes. Subjects with previously diagnosed DM and AD before 1997 were excluded from this study. Newly diagnosed diabetic patients were identified from the cohort database (International Classification of Diseases, Ninth Revision, Clinical Modification [ICD-9-CM] code: 250.xx or ICD-9-A code (abridge code): A181) since January 1, 1997. The identification of DM had been proved valid and used in previous studies [15], [16].

We used propensity scoring to match non-diabetic subjects to diabetic patients, a widely used method for avoiding selection bias in databases with large sample sizes [17]. To balance known risk factors across groups, we considered the following variables including the time when subjects were enrolled, age, gender, hypertension, hyperlipidemia, and previous stroke history.

To investigate whether use of DM medication would affect the course of AD, we evaluated patients’ use of DM medications at baseline (including metformin, sulfonylureas, thiazolidinediones, α-glucosidase blockers, non-sulfonylurea insulin secretagouge, and insulin). These medications were identified and classified by the National Drug Code and the Anatomic Therapeutic Chemical Code, a well-accepted international drug classification system coordinated by the WHO Collaborating Center for Drug Statistics Methodology [18].

### Alzheimer’s Disease Event Measurement

The endpoint of the study was occurrence of administrative claims with AD (ICD-9-CM code: 331.0) as the main diagnosis, either during hospitalization or subsequent outpatient department visits. All the patients were followed up to December 31, 2007. The diagnoses of AD were based on history, physical examination, laboratory and imaging studies, and the Mini-Mental State Examination [19], internationally accepted criteria for AD (National Institute of Neurological and Communicative Disorders and Stroke–Alzheimer’s Disease and Related Disorders Association) [20], and the Diagnostic and Statistical Manual of Mental Disorders [21]. Similar methods for the identification of AD had been applied in our previous study [22].

### Statistical Analysis

Microsoft SQL Server 2005 was used for data management and computing. Statistical analysis was performed utilizing SPSS software (Version 15.0, SPSS Inc., Chicago, IL, USA). All data were expressed as the frequency (percentage) or mean ± standard deviation. The parametric continuous data between the diabetic patients and the non-diabetic subjects were compared by unpaired Student’s *t*-test. The categorical data between the two groups were compared with Chi-square test and Yates’ correction or Fisher’s exact test as appropriate. Survival analysis was assessed using Kaplan-Meier analysis, with the significance based on the log-rank test. The survival time was calculated from the date of DM diagnosis to the date of AD diagnosis. To assess the independent effects of DM, we conducted Cox proportional hazard regression models in all the patients with age, sex, comorbidities (including hypertension, hyperlipidemia, stroke, coronary artery disease, arrhythmia, heart failure, and depression), geographic area, urbanization status, and medications for DM treatment (including metformin, sulfonylureas, thiazolidinediones, α-glucosidase blockers, non-sulfonylurea insulin secretagouge, and insulin) adjusted simultaneously in the model. To assess the independent effects of medications for DM treatment, we conducted Cox proportional hazard regression models in diabetic patients with age, sex, comorbidities (including hypertension, hyperlipidemia, stroke, coronary artery disease, arrhythmia, heart failure, and depression), geographic area, and urbanization status adjusted simultaneously in the model. Statistical significance was inferred at a two-sided *p* value of <0.05.

## Results

A total of 71,433 newly diagnosed diabetic patients (mean age 58.7±14.0 years, female 48.2%) were identified from the 1,000,000 sampling cohort dataset between January 1997 and December 2007. Another 71,311 non-diabetic subjects who were matched using propensity score were enrolled as non-exposure controls. The demographics parameters of study subjects are shown in **Table 1**. Patients with newly diagnosed DM had more coronary artery disease (7.1% vs. 5.5%, *p*<0.001), arrhythmia (3.6% vs. 2.8%, *p*<0.001), heart failure (1.8% vs. 1.0%, *p*<0.001), and depression (0.3% vs. 0.2%, *p* = 0.023) than non-diabetic subjects. The medications used for diabetes treatment for patients with DM included metformin (16.5%), sulfonylureas (74.9%), thiazolidinediones (9.9%), α-glucosidase blockers (9.5%), non-sulfonylurea insulin secretagouge (6.5%), and insulin (18.2%).

**Table 1:** Demographic data of the patients with and without diabetes mellitus.

Variables	Diabetes mellitus				*p*-value
	Yes		No		
	(n = 71,433)		(n = 71,311)		
Age, years	58.7±14.0		58.7±14.0		0.796
Female, n(%)	34,447	(48.2%)	34,369	(48.2%)	0.920
Hypertension, n(%)	16,731	(23.4%)	16,659	(23.4%)	0.788
Hyperlipidemia, n(%)	4,952	(6.9%)	4,862	(6.8%)	0.397
Stroke, n(%)	2,018	(2.8%)	1,939	(2.7%)	0.227
Coronary artery disease, n(%)	5,075	(7.1%)	3,945	(5.5%)	**<0.001**
Arrhythmia, n(%)	2,558	(3.6%)	1,963	(2.8%)	**<0.001**
Heart failure, n(%)	1,311	(1.8%)	722	(1.0%)	**<0.001**
Depression, n(%)	176	(0.3%)	135	(0.2%)	**0.023**
Geographic area, n(%)					**<0.001**
North	32548	(45.6%)	34546	(48.4%)	
Central	12463	(17.4%)	12731	(17.9%)	
South	24352	(34.1%)	22021	(30.9%)	
East	2070	(2.8%)	2013	(2.8%)	
Urbanization status, n(%)					**<0.001**
Metropolis	20149	(28.2%)	21309	(29.9%)	
Satellite city/town	22756	(31.9%)	22712	(31.8%)	
Rural area	28528	(39.9%)	27290	(38.3%)	
Medication, n(%)					
Metformin	4,978	(16.5%)			
Sulfonylureas	22,600	(74.9%)			
Thiazolidinediones	3,001	(9.9%)			
α-glucosidase blockers	2,851	(9.5%)			
Non-sulfonylurea insulin secretagouge	1,955	(6.5%)			
Insulin	5,489	(18.2%)			

Propensity matched for the time when subjects were enrolled, age, gender, hypertension, hyperlipidemia, and previous stroke history.

**Figure 1:**
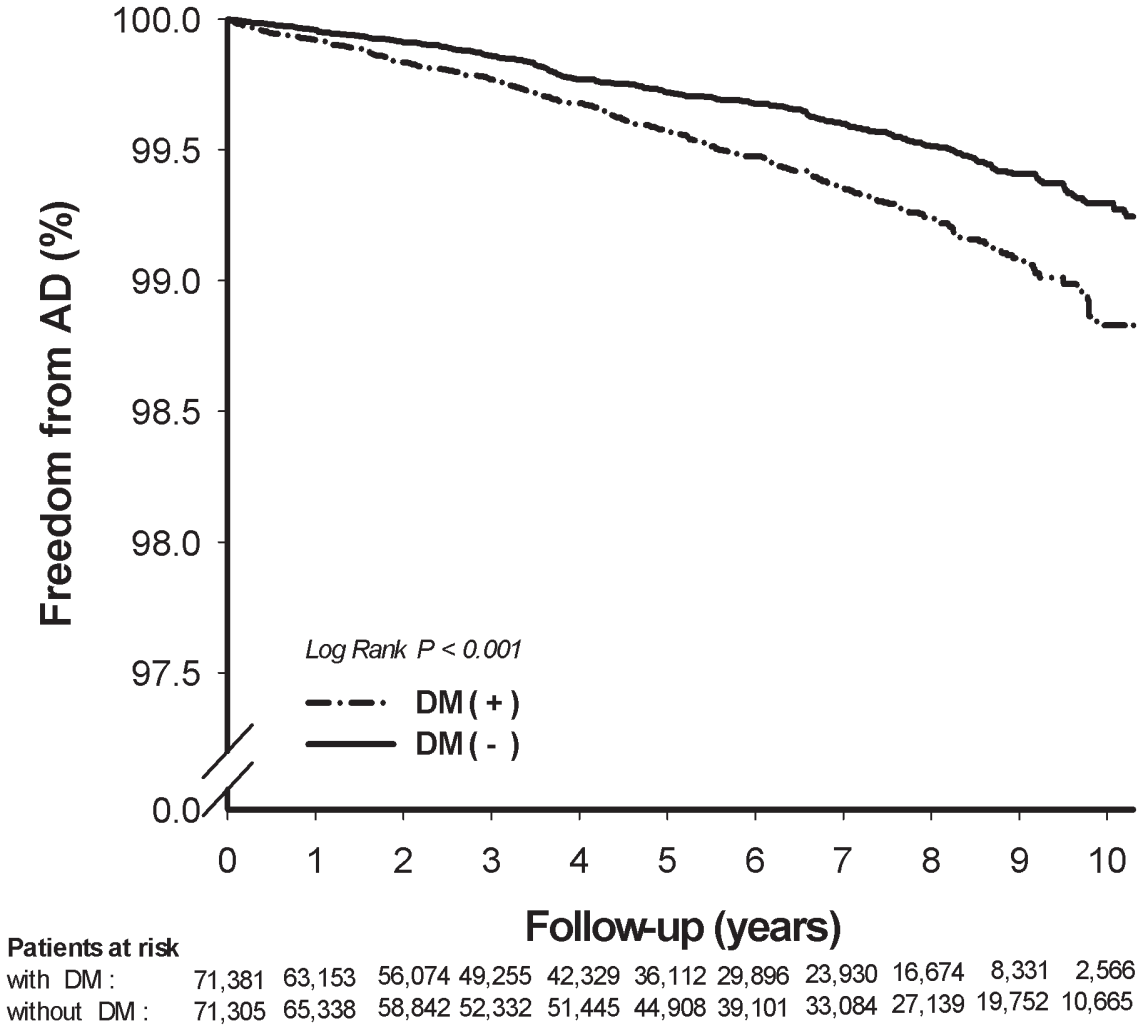
Kaplan-Meier estimates of survival free of Alzheimer’s disease (AD) events in subjects categorized by diabetes mellitus (DM). The event-free survival rates were significantly different in two groups (*p*<0.001 by log rank test).

**Figure 2:**
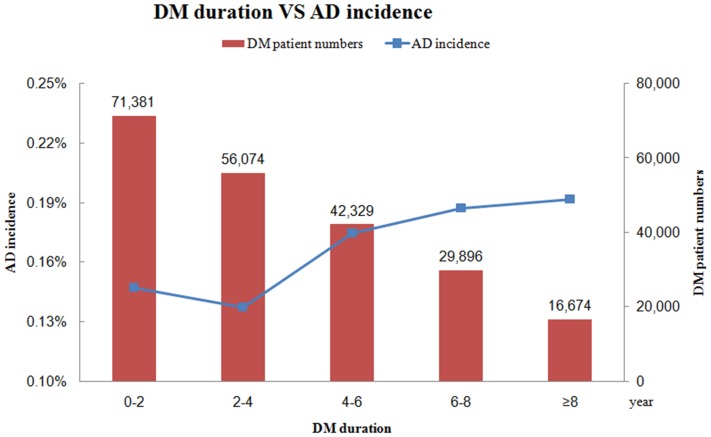
The trend of the incidence of Alzheimer’s disease (AD) according to the duration of diabetes mellitus (DM).

During a maximum 11 years’ follow-up (mean 5.5±3.1 years), 346 (0.48%) of the diabetic patients were diagnosed with AD, and 266 non-diabetic subjects (0.37%) were diagnosed with AD. **Figure 1** exhibits the results of a Kaplan-Meier analysis and the log-rank test showed that diabetic patients had significantly higher incidence of AD than non-diabetic subjects (p<0.001). The risk of developing AD increased gradually in association to longer duration of DM since diagnosis **(** **Figure 2** **)**. To investigate the independent factors associated with the risk of developing AD, Cox regression analysis was performed, with the finding of DM (hazard ratio [HR], 1.76; 95% confidence interval [CI], 1.50–2.07), *p*<0.001), age (HR, 1.11; 95% CI, 1.10–1.12, *p*<0.001), female gender (HR, 1.24; 95% CI, 1.06–1.46, *p* = 0.008), hypertension (HR, 1.30; 95% CI, 1.07–1.59, *p* = 0.01), previous stroke history (HR, 1.79; 95% CI, 1.28–2.50, *p*<0.001), and urbanization status (metropolis, HR, 1.32; 95% CI, 1.07–1.63, *p* = 0.009) were independently associated with the increased risk of AD **(** **Table 2** **)**.

**Table 2:** Independent predictors of Alzheimer’s disease identified by Cox regression analysis.

Variables	HR	(95% CI)	p-value
Diabetes mellitus	1.76	(1.50–2.07)	**<0.001**
Age, years	1.11	(1.10–1.12)	**<0.001**
Female	1.24	(1.06–1.46)	**0.008**
Hypertension	1.30	(1.07–1.59)	**0.010**
Hyperlipidemia	1.06	(0.75–1.51)	0.742
Stroke	1.79	(1.28–2.50)	**<0.001**
Coronary artery disease	0.94	(0.69–1.27)	0.688
Arrhythmia	1.15	(0.78–1.71)	0.475
Heart failure	0.74	(0.40–1.37)	0.342
Depression	1.44	(0.36–5.80)	0.607
Geographic area			
East	1.00		
South	1.42	(0.82–2.45)	0.205
Central	1.18	(0.67–2.09)	0.569
North	1.28	(0.73–2.24)	0.395
Urbanization status			
Rural area	1.00		
Satellite city/town	1.04	(0.83–1.31)	0.703
Metropolis	1.32	(1.07–1.63)	**0.009**

CI = confidence interval, HR = hazard ratio.

Among the 71,433 diabetic patients, there were 2,791 patients with type 1 DM and 68,462 patients with type 2 DM. Both type 1 DM (hazard ratio, 1.89; 95% confident interval, 1.23–2.89, *p* = 0.004) and type 2 DM (hazard ratio, 1.57; 95% confident interval, 1.34–1.85, *p*<0.001) increased the risk of AD.

Medications for DM treatment were analyzed to investigate the relationship between hypoglycemic agents and risk of developing AD in diabetic patients. In initial crude analysis, monotherapy with sulfonylureas (HR, 0.50; 95% CI, 0.34–0.75) was associated with reduced risk of AD. Combination therapy with non-sulfonylurea insulin secretagouge (HR, 2.58; 95% CI, 1.16–5.75), and either monotherapy (HR, 2.27; 95% CI, 1.47–3.51) or combination therapy with insulin (HR, 3.79; 95% CI, 1.89–7.58) were found to be associated with the risk of AD **(** **Table 3** **)**. Neither monotherapy nor combination therapy with oral antidiabetic medications were associated with AD occurrence after adjusting for underlying risk factors and the duration of DM since diagnosis. However, combination therapy with insulin was found to be associated with the greater risk of AD (HR, 2.17; 95% CI, 1.04–4.52, *p* = 0.039).

**Table 3:** Medication for diabetes mellitus and risk of Alzheimer’s disease in diabetic patients.

Medication	Unadjusted HR	Adjusted HR
	(95% CI)	(95% CI)
Metformin: monotherapy	0.88 (0.36–2.16)	0.69 (0.28–1.71)
Metformin: combination therapy	0.60 (0.28–1.30)	0.57 (0.26–1.26)
Sulfonylureas: monotherapy	**0.50 (0.34–0.75)** *	0.75 (0.50–1.13)
Sulfonylureas: combination therapy	0.53 (0.23–1.23)	0.59 (0.25–1.37)
Thiazolidinediones: monotherapy	0.83 (0.12–5.93)	0.92 (0.13–6.60)
Thiazolidinediones: combination therapy	0.51 (0.22–1.17)	0.86 (0.36–2.02)
α-glucosidase blockers: monotherapy	0.88 (0.22–3.58)	0.71 (0.18–2.89)
α-glucosidase blockers: combination therapy	1.11 (0.53–2.34)	1.37 (0.64–2.93)
Non-sulfonylurea insulin secretagouge: monotherapy	1.67 (0.81–3.44)	1.33 (0.64–2.75)
Non-sulfonylurea insulin secretagouge: combination therapy	**2.58 (1.16–5.75)** ‡	2.11 (0.93–4.77)
Insulin: monotherapy	**2.27 (1.47–3.51)** *	1.53 (0.98–2.39)
Insulin: combination therapy	**3.79 (1.89–7.58)** *	**2.17 (1.04**–**4.52)** ‡

CI = confidence interval, HR = hazard ratio.

* *p*<0.001,

† *p*<0.01,

‡ *p*<0.05.

Adjusted for age, sex, comorbidities (including hypertension, hyperlipidemia, stroke, coronary artery disease, arrhythmia, heart failure, and depression), geographic area, and urbanization status.

## Discussion

Our current study revealed that newly diagnosed DM was associated with increased risk of future AD development in this cohort after a maximum of 11 years’ follow-up. Additionally, increasing risk of AD was found to be associated with DM duration, indicating DM maybe an important contribution in the pathogenesis of AD. Furthermore, neither monotherapy nor combination therapy with oral antidiabetic medications were found to be associated with the risk of AD occurrence. However, combination therapy with insulin was found to be associated with the risk of AD occurrence.

The association between DM and AD has been noted in these years. In the Rotterdam Study [8], DM almost doubled the risk of dementia in 6,370 elderly subjects (aged 55 years and older) after average 2.1-year follow up. In the Kungsholmen project [9], DM increases the risk of dementia in 1,301 very old people (aged 75 years and older) in Sweden after 6-year follow-up. In the Canadian Study of Health and Aging [11], however; DM at baseline was associated with incident vascular cognitive impairment but not AD in 5,574 Canadian after 5-year follow-up. Similar results were reported in the Framingham cohort [12] that baseline DM did not increase the risk of incident AD in 2210 participants after 12.7-year follow-up. Different from previous studies [8], [9], [11], [12], our study included patients with newly diagnosed DM from a nationwide cohort dataset in Taiwan. Therefore, the duration of DM was available in our study. This is the largest available database (more than 140,000 subjects) dealing with the relationship between DM and AD risk. Our study results demonstrated diabetic patients carried an increased 1.76 fold risk for AD development, supporting previous studies that DM could be seen as an independent risk factor for incident AD [8], [9]. Furthermore, increasing risk of AD was found to be associated with DM duration in our study, further supporting DM as an important factor influencing in the pathogenesis of AD.

There are some possible mechanisms for the association between DM and AD. First, hyperglycemia may cause increased oxidative stress and accumulation of advanced glycation end-products [23], [24], leading to progressive functional and structural abnormalities in the brain [25]. This hypothesis is further supported by the Hisayama Study [26] conclusion that abnormal response of oral glucose tolerance test after a 75-g oral glucose challenge was closely associated with increased risk of AD, suggesting impaired glucose tolerance contributes to the development of AD. Second, although the cause and progression of AD remains undetermined, β-amyloid peptides deposits are considered as the fundamental cause of the disease. DM is associated with insulin resistance and hyperinsulinaemia, which might interfere with β-amyloid peptides metabolism [27], [28]. Insulin could cross the blood-brain barrier, and the insulin levels in brain are initially higher and then down-regulated in diabetic patients [29]. Since insulin may modulate β-amyloid peptides degradation by regulating expression of the insulin-degrading enzyme [27], the low insulin level in central nervous system may reduce insulin-degrading enzyme levels in brain and thereby impair β-amyloid peptides clearance. The aggregation of β-amyloid peptides is a fundamental neuropathological hallmark of AD.

Since DM has been reported to be associated with AD, therapeutic strategies aim at treating DM is a topic of interest for avoiding AD development [30], [31]. In the Rotterdam Study, diabetic patients treated with insulin were at highest risk of dementia [8]. In the Kungsholmen project [9], patients being treated with oral antidiabetic medications had increased risk for dementia and vascular dementia. It has been suggested that metformin, the most widely used insulin sensitizer against peripheral insulin resistance, could sensitize neuronal insulin resistance and significantly improved AD-like changes [32]. Wu et al. [33] reported that antidiabetic medications appear to be useful in alleviating the decline in physical and cognitive functioning among older Mexican Americans with DM, especially for those with a longer duration of the disease. Beeri et al. [34] reported that the combination of insulin with other oral antidiabetic medications is associated with substantially lower neuritic plaque density consistent with the effects of both on the neurobiology of insulin. The association between AD and hypoglycemic agent is inconsistent and still remains controversial [8], [9], [30]–[34]. In the crude analysis of our study, decreased AD risk was found to be associated with monotherarpy with sulfonylurea; increased risk for AD was associated with combination therapy using non-sulfonylurea insulin secretagouge, and either monotherapy or combination with insulin. Neither monotherapy nor combination therapy with oral antidiabetic medications were found to be associated with the risk of AD after adjustment for underlying risk factors and the duration of DM since diagnosis. This finding suggests DM or underlying comorbidities, not hypoglycemic agent, are more important determinants of future risk of developing AD. Similar observations show that insulin sensitizers may have beneficial effects on AD, and these benefits may be offset later by longer exposure to DM [30], [31], further supporting the idea that duration of diabetes may play an important role in AD pathogenesis. However, combination therapy with insulin was found to be associated with greater risk of AD. This observation is compatible with the Rotterdam Study that found diabetic patients treated with insulin were at the highest risk for dementia. Since combination therapy with insulin may represent greater severity of DM, these patients were at increased risk for AD.

Another important issue is whether antidiabetic medications prolong the life of the patients with AD. In our current study, we failed to find the beneficial effects of insulin or oral antidiabetic medications in prolonging the life of the patients who developed AD (data not shown). However, it was not the goal of our study and the data is limited to the small sample size of the patients with AD. Further studies are still needed to answer this question.

In addition to DM, the incidence of AD was independently associated with age, female gender, hypertension, and previous history of stroke in our study. Our findings were compatible with previous studies that age and female gender were risk factors for AD occurrence [35]. Similar to previous studies [36], we also found that vascular risk factors including hypertension and previous history of stroke were related to an increased risk of AD.

The main strength of our study is the use of a population-based dataset, which enrolls large sample-size subjects and enables us to trace prospectively the differences between the two groups. However, there are still some limitations in our study. First, the diagnosis of DM was identified using the ICD-9 code from the database. This study was conducted with the NHIRD, in which the diagnosis was supposed to be confirmed clinically by the individual physicians in charge. The identification of DM had been proved valid and used in previous studies [15], [16]. Furthermore, the control group was selected from those patients who didn’t develop diabetes over the whole study periods (maximum of up to 11 years). Therefore, the possibility of underestimating of undiagnosed diabetes and prediabetes could be minimized. Second, the diagnosis of AD was identified using the ICD-9 code from both inpatient and outpatient database. Although the database doesn’t contain detailed information, such as dementia rating scale [37], diagnoses of AD are usually made based on history, physical examination, imaging studies, and quantitative functional scale tools such as the Mini-Mental State Examination [19] as well as other well-known criteria for AD diagnosis (National Institute of Neurological and Communicative Disorders and Stroke–Alzheimer’s Disease and Related Disorders Association) [20]. Similar methods for the identification of AD had been applied in previous studies [22]. Third, personal information such as body mass index, education, smoking habit and biochemistry profiles were not available in the database. Fourth, measurements indicating severity of DM, including serum concentration of Hemoglobin A1c, glucose and insulin, were not available. However, information about the medications taken by diabetes patients was clear and confirmed. Therefore, we can still investigate the effect of the medication. Finally the data regarding APOE4 genotype was also not available in the NHIRD dataset.

## Conclusions

The present study demonstrates an association between DM and future development of AD, suggesting that DM could play an important role in determining future risk of AD occurrence. However, we found that use of hypoglycemic agents had no beneficial effects for preventing development of AD. Further therapeutic strategies should be investigated for the prevention of AD, such as preventing DM or improving DM treatment.
